# Assessment of blood one-carbon metabolism indexes during mid-to-late pregnancy in 397 Chinese pregnant women

**DOI:** 10.3389/fnut.2024.1348930

**Published:** 2024-02-08

**Authors:** Rong Zhang, Xiangyi Wu, Lu Lu, Rui Hu, Yue Teng, Lina Pan, Xiaoling Zeng, Wei Jiang, Wei Li, Ling Dong, Wenli Zhu

**Affiliations:** ^1^Department of Nutrition and Food Hygiene, School of Public Health, Health Science Centre, Peking University, Beijing, China; ^2^Gaomi City People's Hospital, Weifang, China; ^3^Beijing Huairou Maternity and Child Health Care Hospital, Beijing, China; ^4^Haidian Maternal and Child Health Hospital of Beijing, Beijing, China; ^5^Hunan Ausnutria Institute of Food and Nutrition, Changsha, China

**Keywords:** one-carbon metabolism (OCM), pregnancy, folate, betaine, homocysteine (HCY), Sadenosylmethionine (SAM)

## Abstract

**Objectives:**

One-carbon metabolism (OCM) significantly influences fetal growth and neurodevelopment through transferring methyl group to biomolecules, during which folate, methionine, choline and betaine function as methyl donor nutrients, while vitamin B_2_, B_6_, B_12_ function as enzyme cofactors, and homocysteine (Hcy) and S-adenosyl methionine (SAM) are functional metabolites. This study aimed to assess blood OCM index levels and explore their relationships among Chinese pregnant women.

**Methods:**

Data were obtained from the baseline of the Mother–Child Nutrition and Health Cohort Study. Pregnant women, voluntarily recruited from September 2020 to June 2022 during antenatal examinations in five Chinese cities at 24–32 gestational weeks, provided fasting venous blood samples. Measurements included RBC and serum folate, serum vitamin B_2_, B_6_, B_12_, choline, betaine, methionine, total Hcy (tHcy), and plasma SAM. Sociodemographic characteristics and pregnancy-related conditions were collected via a self-designed questionnaire.

**Results:**

Of 397 participants, 82.6% were in mid-pregnancy (24–27 gestational weeks) and 17.4% were in late-pregnancy (28–32 gestational weeks). Serum folate, vitamin B_6_, and B_12_ deficiencies were 2.5, 1.3, and 8.3%, respectively. Elevated tHcy (≥10 μmol/L) was observed in 1.8% of pregnant women. Elderly pregnant women (aged 35 and above) exhibited significantly lower serum methionine levels (*p* < 0.05), while multiparous women had lower RBC folate levels (*p* < 0.05), and lower serum methionine and vitamin B_12_ levels (*p* < 0.10, not statistically significant). Partial correlation analysis revealed positive associations between RBC folate and cofactor vitamin B_12_ (*r* = 0.244, *p* < 0.05) in the folate cycle, as well as significant correlations between two methyl donor paths [serum folate was significantly related to serum choline (*r* = 0.172) and betaine (*r* = 0.193)]. As functional biomarkers of OCM, serum tHcy exhibited negative associations with RBC folate (*β* = −0.330, *p* < 0.05) and vitamin B_6_ (*β* = −0.317, *p* < 0.05), and plasma SAM displayed a positive association with serum betaine (*β* = 0.610, *p* < 0.05), while negatively associated with serum vitamin B_6_ (*β* = −0.181, *p* < 0.05).

**Conclusion:**

The blood OCM exhibited imbalances during mid-to-late pregnancy, characterized by lower levels of folate, vitamin B_6_, and B_12_, alongside elevated tHcy levels. Adequate folate and vitamin B_6_ emerged as significant predictors of lower tHcy levels. Additionally, serum betaine showed a positive correlation with plasma SAM. This suggests the importance of not only ensuring sufficient folate but also optimizing other OCM-related nutrients throughout pregnancy.

## 1 Introduction

One-carbon metabolism (OCM) supplies one-carbon (1C) units necessary for DNA, proteins, and lipids biosynthesis, as well as for the epigenetic modification of the genome. It plays a critical role in cell proliferation and gene expression, particularly in cell growth process such as embryonic development and malignant tumors (1–3). The prenatal period is characterized by rapid cellular proliferation and differentiation, and epigenetic marks established early in life can be maintained and exert lasting impacts on gene expression and functions later in life. Extensive research has explored the impact of maternal OCM during pregnancy on early development and fetal programming. It has been demonstrated that imbalances in prenatal 1C nutrients (deficiency/excess) can impede fetal growth, neurodevelopment, and cardiometabolic parameters in childhood (4). Furthermore, they have been associated with childhood allergies (5–7).

OCM encompasses the folate cycle and the methionine cycle, involving various nutrients, such as B vitamins, amino acids, minerals and carbohydrates. Among these, folate, choline, betaine and methionine serve as methyl donors; while vitamin B_2_, B_6_, B_12_ act as coenzymes, and S-adenosylmethionine (SAM) and homocysteine (Hcy) serve as functional biomarkers of OCM (2). These nutrients and their metabolites interact in a complex manner. In fact, despite the supplementation of folic acid during periconception has been recommended worldwide since the 1990s (8), studies have shown that folate deficiency still existed (9–11). This emphasizes the necessity not only for folate but also for a comprehensive approach to OCM.

The interplay among OCM nutrients holds significance. Animal studies reported that methyl donors regulated metabolism, immune response, and epigenetic events through interactions within the OCM (12). A review suggested that reduced folate levels were associated with enhanced choline oxidation pathway activity during pregnancy; the roles of nutrients such as folate and cobalamin were closely linked and their imbalance has been associated with an increased risk of adverse pregnancy outcomes (13). Plasma total homocysteine (tHcy), an intermediate product of methionine metabolism, might serve as an independent risk factor for cognitive decline, irrespective of B-vitamin deficiency (14). The Reus-Tarragona Birth Cohort revealed that during 24–27 gestational weeks, both plasma folate and betaine exhibited an inverse association with plasma tHcy. Furthermore, the association between betaine and tHcy was dependent on folate status. Another study indicated that blood betaine showed an inverse association with tHcy at labor, irrespective of folate status (15). The precise interactions among OCM indexes remained unclear.

Moreover, while numerous studies have concentrated on dietary intake of OCM nutrients and their effects, data on circulating OCM nutrient/metabolite status and their interactions remain limited. In fact, the levels of blood OCM indices result from an interaction between dietary intake and genetic factors, offering a precise reflection of the OCM status. Perturbations in OCM might establish a significant link between early environmental exposure and later-life offspring development (2, 16). Studies have revealed imbalances in blood OCM during pregnancy, yet uncertainties persist regarding their cutoff values and associated factors (17–19). A case–control study found that pregnant women who supplemented folic acid throughout pregnancy had higher serum folate concentrations compared to those who took supplementation during short periods (20). Maternal serum tHcy was positively associated with maternal age (21), and negatively with pregnancy-associated plasma protein A (22).

Therefore, our study aimed to examine the levels of blood OCM indices among Chinese pregnant women to delineate their distribution, interactions, and associated factors.

## 2 Materials and methods

### 2.1 Participants

Data was obtained from the baseline of the Mother–Child Nutrition and Health Cohort Study (MCNHC), which followed mother–child pairs during gestation and the postnatal period until age 7 years, in five cities (Beijing, Qingdao, Gaomi, Changsha and Zhuzhou) located in east and central China.

At baseline, pregnant women were voluntarily recruited during their antenatal examination visits at local maternal and child care clinics, from September 2020 to June 2022. The inclusion criteria of participants were as follows: (a) Pregnant women at 24–32 gestational weeks; (b) Having a single pregnancy; and (c) Residing in the local region for more than 5 years. The research clinicians explained the study protocol to all included pregnant women, finally, 63.9% provided their consent and 800 participants were enrolled in the cohort voluntarily. Women with such situations were excluded: (a) Using anti-folate drugs; (b) Experiencing abnormal fetal intrauterine development; (c) Having serious gestational complications.

In the study the blood measurements were detected in half of the participants (*n* = 397). The sample size was deemed sufficient for drawing conclusions based on existing literature (22–24).

The study received approval from the Peking University Institutional Review Board (Beijing, China, approval number IRB00001052-19145), and was conducted according to the Declaration of Helsinki. Written informed consent was obtained from all pregnant participants, guaranteeing the protection of their privacy and confidential personal information.

### 2.2 Blood OCM measurement and data collection

**Figure 1:**
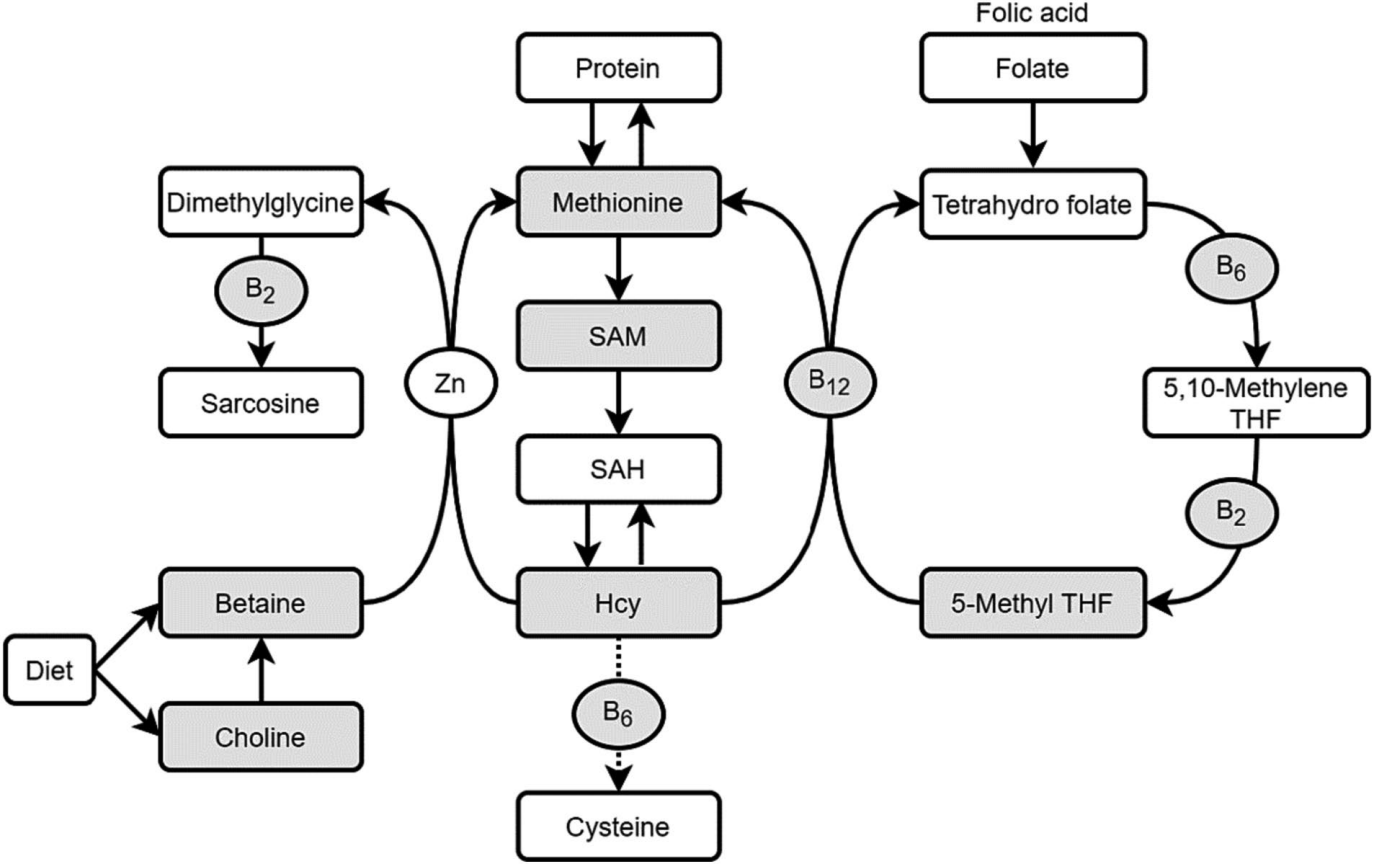
One-carbon metabolism (OCM) nutrients and metabolites. THF, Tetrahydro folate; Hcy, homocysteine; SAM, S-adenosyl methionine; SAH, S-adenosyl homocyateine.

Fasting venous blood samples of 397 pregnant women at 24–32 gestational weeks were collected to measure OCM nutrients and metabolites (folate, vitamin B_2_, B_6_, B_12_, choline, betaine, methionine, homocysteine and S-adenosyl methionine, the shaded indicators as shown in Figure 1). Blood samples were obtained in various forms, including non-anticoagulated blood, as well as anticoagulated blood using EDTA and heparin, respectively.

The blood samples were processed differently based on their anticoagulant, with EDTA-anticoagulated blood stored in a 4°C ~ 8°C refrigerator until the measurement of red blood cell folate (RBC folate) using microparticle chemiluminescence. Heparin-anticoagulated blood was utilized to determine plasma SAM via liquid phase tandem mass spectrometry. For the remaining OCM indexes, non-anticoagulated blood was employed, and measurements were conducted using various methods: serum folate by microparticle chemiluminescence, tHcy using the enzyme cycling method, serum vitamin B_12_ via chemiluminescence, and serum vitamin B_2_, B_6_, choline, betaine, and methionine by liquid phase tandem mass spectrometry.

Finally, the serum folate, tHcy, vitamin B_2_, vitamin B_6_, vitamin B_12_, choline, and betaine concentrations were valid for all 397 participants. However, due to the excluded hemolyzed samples, the valid sample size for RBC folate and plasma SAM were 378 and 277, respectively. Serum methionine was detected in half of the participants (*n* = 196). The sample sizes for these indicators were deemed adequate for drawing scientifically valid conclusions based on relevant literature (11, 20, 22).

A self-designed questionnaire was employed to gather information on sociodemographic characteristics such as age, educational level, employment status, and household income. Additionally, it delved into pregnant conditions, encompassing gestational weeks, pregnancy and labor histories, and nutrient supplementation during pregnancy. The questionnaire was self-administered online, with trained investigators available to offer explanations following a standardized procedure manual whenever required. Upon completion, the questionnaire underwent a thorough check, and any missing information was verified. Ultimately, valid data was obtained from all 397 participants.

### 2.3 Statistical analysis

Data organization and statistical analyses were performed utilizing SPSS software (version 27.0, IBM Corp, Armonk, NY, United States).

The criteria for defining deficiencies in various OCM indicators were established based on established standards: folate deficiency was characterized as RBC folate <151 ng/mL and/or serum folate <10 nmol/L following WHO standards (25). Vitamin B_12_ deficiency was defined as≤148 pmol/L (26), while vitamin B_6_ deficiency was defined as serum B_6_ < 4.94 ng/mL according to the CDC’s Second National Report on Biochemical Indicators of Diet and Nutrition in the US Population (27). Hyperhomocysteinemia (HHcy) was classified as tHcy ≥10 μmol/L (28). No recognized cutoffs were available for other OCM indicators.

SPSS software was used to analyze the data. The normal distribution of OCM indexes was assessed using the K-S test. Descriptive statistics were applied to present serum folate, choline, betaine, and methionine concentrations as Mean ± SD, while RBC folate, B vitamins, tHcy, and SAM concentrations were depicted using medians and quartiles. Group comparisons of OCM indexes were conducted utilizing t-tests, one-way ANOVA, LSD test, and Mann–Whitney U test as appropriate. Correlations among OCM indexes were examined through Pearson Correlation Analysis. Furthermore, multiple linear regression was employed to explore the relationships between functional biomarkers (tHcy, SAM) and other OCM indicators. A statistical significance level of 0.05 was set for all analyses.

## 3 Results

### 3.1 Characteristics of participants

Of the participants, 82.6% were in mid-pregnancy (24–27 gestational weeks), while 17.4% were in late-pregnancy (28–32 gestational weeks). Around 33.0% of the participants were over 35 years old, and nearly half of them reported having attained a bachelor’s degree or higher education. Approximately two-thirds (61.0%) of the participants took nutrient supplements during pregnancy, with 52.4% of them including folate in their supplements. Further details are available in Table 1.

**Table 1:** Characteristics of participants (*N* = 397).

Characteristics	n	Percentage (%)
Age (years)		
18 ~ 29	101	25.4
30 ~ 34	165	41.6
35 ~ 46	131	33.0
Educational level		
≤High school	108	27.2
Junior college	111	28.0
≥Bachelor	178	44.8
Gestational weeks		
24 ~ 27	328	82.6
28 ~ 32	69	17.4
Employment		
Employed	288	72.5
Unemployed	109	27.5
Household income *per capita* monthly (yuan)		
≤6,000	68	17.1
6,001 ~ 10,000	156	39.3
≥10,001	173	43.6
Number of pregnancies		
1	162	40.8
≥2	235	59.2
Number of deliveries		
0	221	55.7
≥1	176	44.3
Nutrient supplementation		
With folic acid	208	52.4
Without folic acid	74	18.6
No supplementation	115	29.0

### 3.2 Distribution of blood OCM index levels during pregnancy

The median RBC folate level was 1032.83 (*P_25_*-*P_75_*: 727.45 ~ 1180.84) ng/mL, showing no RBC folate deficiency (<151 ng/mL). The median serum folate was 32.13 (*P_25_*-*P_75_*: 20.55 ~ 46.69) nmol/L with 2.5% experiencing serum folate deficiency (<10 nmol/L). Notably, 8.3% of pregnant women showed vitamin B_12_ deficiency (<148 pmol/L) while 1.8% had increased tHcy (≥10 μmol/L). The median level of vitamin B_6_ was 16.50 (*P_25_*-*P_75_*: 10.65 ~ 24.25) ng/mL, with 1.3% exhibiting vitamin B_6_ deficiency (< 4.94 ng/mL). As shown in Table 2.

**Table 2:** Blood OCM index levels of the pregnant women.

Blood OCM^*^	n	*Mean* ± *SD*	*Median* (*P_25_*-*P_75_*)	Abnormal n (%)
RBC folate (ng/mL)	378	968.39 ± 287.94	1032.83 (727.45 ~ 1180.84)	0 (0.0)^a^
Serum folate (nmol/L)	397	32.83 ± 14.04	32.13 (20.55 ~ 46.69)	10 (2.5)^a^
Serum tHcy^*^ (μmol/L)	397	5.38 ± 10.25	4.78 (3.90 ~ 5.55)	7 (1.8)^b^
Serum Vitamin B_2_ (ng/mL)	397	8.07 ± 9.39	4.75 (2.50 ~ 9.46)	—
Serum Vitamin B_6_ (ng/mL)	397	18.01 ± 9.26	16.50 (10.65 ~ 24.25)	5 (1.3)^a^
Serum Vitamin B_12_ (pmol/L)	397	234.26 ± 75.12	223.00 (184.00 ~ 268.00)	33 (8.3)^a^
Serum Choline (μmol/L)	397	9.98 ± 2.95	9.70 (8.33 ~ 11.37)	—
Serum Betaine (μmol/L)	397	29.29 ± 14.25	26.00 (20.05 ~ 37.10)	—
Plasma SAM^*^(nmol/L)	277	56.10 ± 19.20	52.50 (43.50 ~ 65.60)	—
Serum Methionine (μmol/L)	196	24.01 ± 5.73	23.80 (19.90 ~ 27.30)	—

^a^Deficiency; ^b^Excess; ^*^OCM, one-carbon metabolism; SAM, S-adenosylmethionine; tHcy, total homocyateine.

Individual and household characteristics, and gestational history were associated with the pregnant blood OCM index levels, as shown in Tables 3, 4. The results showed education level and household income were significantly related to folate, vitamin B_2_, vitamin B_6_ and choline levels, higher the educational level and household income of pregnant women, higher the levels of RBC folate, choline and vitamin B_2_ (*p* < 0.05). Pregnant women aged 35 and above had notably lower serum methionine levels (*p* < 0.05). Additionally, compared to primiparous women, multiparous women exhibited lower RBC folate levels (*p* < 0.05), serum methionine, and vitamin B_12_ levels (*p* < 0.10, but with no significance).

**Table 3:** Blood methyl-donor nutrients levels according to participants’ characteristics, Mean ± SD.

Group	RBC folate (ng/mL) *Median* (*P_25_–P_75_*)	Serum folate (nmol/L)	Serum choline (nmol/L)	Serum betaine (μmol/L)	Serum methionine (μmol/L)
Age (years)					
18 ~ 29	1044.87 (728.60 ~ 1176.41)	30.55 ± 13.48	10.09 ± 2.81	30.80 ± 14.76	25.48 ± 6.19^a^
30 ~ 34	1001.71 (714.83 ~ 1174.94)	33.72 ± 13.45	9.70 ± 2.85	28.27 ± 13.06	24.32 ± 5.87^a^
35 ~ 46	1017.77 (716.14 ~ 1198.09)	32.89 ± 14.89	10.19 ± 3.18	28.82 ± 14.82	22.56 ± 5.11^b^
*P*	0.847	0.199	0.342	0.359	0.023*
Gestational weeks					
24 ~ 27	1042.79 (715.21 ~ 1187.75)	32.66 ± 13.97	10.03 ± 2.91	29.43 ± 14.50	23.80 ± 5.65
28 ~ 31	988.80 (772.63 ~ 1136.70)	32.49 ± 14.17	9.65 ± 3.19	27.57 ± 12.09	24.62 ± 6.31
*P*	0.465	0.928	0.327	0.321	0.441
Educational level					
≤High school	997.44 (691.16 ~ 1175.87)^a^	31.48 ± 13.08	9.37 ± 2.16^a^	29.35 ± 13.44	24.08 ± 6.60
Junior college	1019.68 (697.56 ~ 1165.54)^a^	32.38 ± 14.75	10.09 ± 2.85	29.53 ± 13.67	24.11 ± 5.78
≥Bachelor	1096.46 (836.94 ~ 1213.69)^b^	33.42 ± 14.10	10.26 ± 3.37^b^	28.76 ± 14.83	23.80 ± 5.35
*P*	0.031*	0.524	0.042*	0.890	0.938
Employment					
Employed	1003.09 (710.24 ~ 1177.73)	32.23 ± 14.06	9.94 ± 3.06	29.54 ± 14.30	24.04 ± 5.65
Unemployed	1045.95 (755.08 ~ 1193.28)	33.58 ± 13.86	10.05 ± 2.68	28.08 ± 13.62	23.72 ± 6.12
*P*	0.618	0.393	0.752	0.362	0.731
Household income *per capita* monthly (yuan)					
≤6,000	996.13 (818.28 ~ 1209.11)^a^	31.25 ± 14.62	9.58 ± 2.96^a^	27.32 ± 14.24	24.34 ± 6.26
6,001 ~ 10,000	1008.44 (694.55 ~ 1185.52)^b^	31.64 ± 14.16	9.80 ± 2.78	28.40 ± 12.48	24.12 ± 6.29
≥10,001	1040.71 (728.60 ~ 1171.22)^b^	34.09 ± 13.56	10.25 ± 3.10^b^	30.46 ± 15.36	23.67 ± 5.13
*P*	0.046*	0.097	0.049*	0.223	0.824
Number of pregnancies					
1	1067.12 (754.45 ~ 1191.84)	33.25 ± 13.48	9.96 ± 2.92	29.48 ± 14.76	24.29 ± 5.10
≥2	993.85 (710.24 ~ 1176.41)	32.20 ± 14.34	9.97 ± 2.99	28.84 ± 13.67	23.71 ± 6.21
*P*	0.035*	0.468	0.972	0.664	0.494
Number of deliveries					
0	1045.68 (730.45 ~ 1185.85)	32.46 ± 13.71	10.10 ± 2.94	29.40 ± 13.91	24.41 ± 5.26
≥1	999.11 (706.01 ~ 1176.41)	32.83 ± 14.37	9.80 ± 2.97	28.86 ± 14.30	23.38 ± 6.33
*P*	0.042*	0.797	0.319	0.708	0.077
Nutrient supplementation					
With folic acid	1036.37 (735.99 ~ 1185.15)	32.75 ± 13.77	9.66 ± 2.84	29.31 ± 13.10	24.24 ± 6.07
Without folic acid	1064.42 (728.95 ~ 1178.96)	34.82 ± 14.35	10.20 ± 2.79	27.79 ± 12.65	24.32 ± 5.36
No supplement	996.14 (707.92 ~ 1172.95)	31.40 ± 14.16	10.41 ± 3.25	30.00 ± 16.72	23.35 ± 5.42
*P*	0.931	0.269	0.078	0.580	0.614

*p < 0.5; ^a, b^mean significant difference among groups.

**Table 4:** Blood OCM cofactors and functional markers levels according to participants’ characteristics, Median *(P_25_–P_75_)*.

Group	Serum vitamin B_2_ (ng/mL)	Serum vitamin B_6_ (ng/mL)	Serum vitamin B_12_ (ng/mL)	Serum tHcy (μmol/L)	Plasma SAM (nmol/L)
Age (years)					
18 ~ 29	4.90 (2.69 ~ 10.15)	16.05 (11.10 ~ 23.38)	217.50 (179.50 ~ 268.75)	4.85 (3.78 ~ 5.59)	52.50 (44.10 ~ 62.00)
30 ~ 34	4.62 (2.41 ~ 10.50)	16.90 (11.30 ~ 24.20)	218.00 (182.00 ~ 269.00)	4.66 (3.86 ~ 5.67)	53.40 (43.40 ~ 68.30)
35 ~ 46	4.25 (2.49 ~ 8.93)	16.05 (9.82 ~ 24.33)	225.50 (185.50 ~ 266.00)	4.80 (4.07 ~ 5.41)	51.90 (42.48 ~ 67.33)
*P*	0.497	0.812	0.686	0.768	0.678
Gestational weeks					
24 ~ 27	4.87 (2.49 ~ 9.79)	16.65 (11.03 ~ 24.38)	224.00 (185.25 ~ 265.00)	4.80 (3.88 ~ 5.59)	52.70 (43.65 ~ 65.70)
28 ~ 31	3.80 (2.50 ~ 8.82)	15.00 (9.32 ~ 22.65)	212.00 (176.50 ~ 289.50)	4.72 (3.93 ~ 5.31)	50.10 (43.05 ~ 62.70)
*P*	0.463	0.183	0.865	0.677	0.653
Educational level					
≤High school	4.12 (2.33 ~ 8.21)^a^	16.65 (11.08 ~ 22.78)	220.50 (182.75 ~ 264.50)	4.80 (3.98 ~ 5.55)	50.80 (43.90 ~ 60.70)
Junior college	4.77 (2.68 ~ 9.20)^ab^	16.00 (10.70 ~ 25.30)	224.00 (184.00 ~ 269.00)	4.80 (3.96 ~ 5.67)	51.00 (42.60 ~ 65.40)
≥Bachelor	5.09 (2.52 ~ 11.0)^b^	16.50 (10.50 ~ 23.60)	225.00 (185.00 ~ 268.00)	4.75 (3.80 ~ 5.42)	53.40 (43.90 ~ 66.55)
*P*	0.048*	0.905	0.843	0.444	0.725
Employment					
Employed	4.59 (2.44 ~ 9.57)	16.15 (10.70 ~ 23.30)	221.50 (182.25 ~ 269.00)	4.83 (3.95 ~ 5.62)	53.40 (43.85 ~ 66.00)
Unemployed	4.80 (2.69 ~ 9.44)	16.75 (10.63 ~ 25.15)	224.50 (187.00 ~ 263.00)	4.59 (3.82 ~ 5.33)	50.70 (42.50 ~ 61.90)
*P*	0.469	0.767	0.602	0.086	0.404
Household income *per capita* monthly (yuan)					
≤6,000	3.70 (2.53 ~ 6.87)^a^	14.95 (10.07 ~ 22.05)^a^	222.00 (170.00 ~ 258.00)	4.51 (3.69 ~ 5.34)	51.20 (47.35 ~ 58.90)
6,001 ~ 10,000	4.96 (2.45 ~ 10.9)^b^	16.00 (9.93 ~ 24.10)^b^	217.00 (184.00 ~ 265.00)	4.80 (4.04 ~ 5.51)	51.50 (42.70 ~ 65.35)
≥10,001	5.18 (2.55 ~ 8.63)^b^	18.00 (11.90 ~ 25.50)^c^	225.00 (188.25 ~ 274.75)	4.81 (3.94 ~ 5.67)	54.45 (43.93 ~ 67.00)S
*P*	0.035*	0.018*	0.391	0.435	0.579
Number of pregnancies					
1	4.75 (2.32 ~ 9.30)	15.90 (10.80 ~ 23.30)	226.00 (187.00 ~ 273.00)	4.78 (3.95 ~ 5.43)	52.60 (44.10 ~ 65.70)
≥2	4.67 (2.66 ~ 9.53)	16.80 (10.68 ~ 24.70)	220.00 (181.75 ~ 263.50)	4.79 (3.86 ~ 5.67)	52.05 (42.60 ~ 65.43)
*P*	0.867	0.494	0.089	0.720	0.450
Number of deliveries					
0	4.75 (2.36 ~ 9.30)	16.10 (10.80 ~ 23.30)	226.00 (184.00 ~ 273.00)	4.80 (3.97 ~ 5.51)	52.50 (43.58 ~ 65.43)
≥1	4.67 (2.64 ~ 9.53)	16.55 (10.58 ~ 24.85)	221.50 (180.50 ~ 260.75)	4.69 (3.79 ~ 5.64)	52.20 (43.58 ~ 66.00)
*P*	0.928	0.700	0.622	0.639	0.963
Nutrient supplementation					
With folate	4.84 (2.43 ~ 9.02)	15.85 (10.45 ~ 23.28)	229.50 (187.25 ~ 271.25)	4.72 (3.93 ~ 5.54)	53.50 (43.55 ~ 66.80)
Without folate	4.96 (2.69 ~ 13.2)	18.30 (11.60 ~ 25.50)	216.00 (188.50 ~ 272.00)	4.86 (3.85 ~ 5.38)	50.75 (44.55 ~ 63.58)
No supplement	4.46 (2.59 ~ 9.84)	16.40 (10.70 ~ 25.00)	216.00 (168.00 ~ 251.00)	4.72 (3.82 ~ 5.59)	52.15 (45.08 ~ 65.03)
*P*	0.419	0.399	0.077	0.965	0.811

*p < 0.5; ^a, b^mean significant difference among groups. OCM, one-carbon metabolism; SAM, S-adenosylmethionine; tHcy, total homocysteine.

### 3.3 Correlation between blood OCM nutrients and metabolites

The study involved the detection of methyl donor nutrients (folate, choline, betaine, methionine), cofactors (vitamin B_2_, B_6_, B_12_), and functional biomarkers (tHcy, SAM) within the OCM pathway, with their interactions explored through correlation analysis.

The findings revealed several significant correlations within OCM indexes. In the folate cycle, RBC folate demonstrated positive associations with serum folate (*r* = 0.336, *p* < 0.05) and cofactor vitamin B_12_ (*r* = 0.244, *p* < 0.05). Serum folate exhibited connections with other methyl donor nutrients, including serum choline (*r* = 0.172, *p* < 0.05) and betaine (*r* = 0.193, *p* < 0.05). As a functional marker of OCM, plasma SAM displayed a significant relationship with serum betaine (r = 0.627, *p* < 0.05). Unexpectedly, vitamin B_6_ showcased negative correlations with SAM (*r* = −0.296, *p* < 0.05) and betaine (*r* = −0.172, *p* < 0.05), as illustrated in Table 5.

**Table 5:** Partial correlation coefficients between OCM nutrients and metabolites.

Blood OCM indexes	Serum folate	RBC folate	Serum choline	Serum betaine	Serum methionine	Serum vitamin B_2_	Serum vitamin B_6_	Serum vitamin B_12_	Serum tHcy
RBC folate	0.336*	—	—	—	—	—	—	—	—
Serum Choline	0.172*	0.032	—	—	—	—	—	—	—
Serum Betaine	0.193*	0.108	−0.104	—	—	—	—	—	—
Serum Methionine	−0.026	0.094	−0.056	0.131	—	—	—	—	—
Serum vitamin B_2_	0.128	0.072	−0.042	0.096	0.064	—	—	—	—
Serum vitamin B_6_	−0.011	0.056	−0.134	−0.172*	−0.037	0.171*	—	—	—
Serum vitamin B_12_	0.091	0.244*	0.029	−0.051	0.040	−0.121	0.004	—	—
Serum tHcy	0.040	0.002	0.047	−0.032	0.011	−0.045	0.041	−0.050	—
Plasma SAM	0.109	0.077	−0.116	0.627*	−0.012	−0.014	−0.296*	−0.059	−0.070

Adjustment of age. *p < 0.05.

### 3.4 Predictors of OCM functional biomarkers

Regarding the principal functional metabolic biomarkers, multiple linear regression analysis of serum tHcy highlighted educational level, blood folate and vitamin B_6_ as significant predictors of lower tHcy level. When RBC folate was utilized as the independent variable (model 1), educational level (*β* = −0.216, *p* < 0.05), RBC folate (*β* = −0.330, *p* < 0.05) and vitamin B_6_ (*β* = −0.317, *p* < 0.05) displayed negative associations with tHcy levels, as shown in Table 6.

**Table 6:** Multiple linear regression analysis of serum tHcy of pregnant women.

Independent variable	Model 1			Model 2		
	*B*	*β*	*p*	*B*	*β*	*p*
Age	1.970	0.095	0.275	1.756	0.090	0.268
Gestational weeks	0.206	0.005	0.949	0.158	0.042	0.933
Educational level	−3.961	−0.216	0.020*	−3.778	−0.213	0.015*
Employment	−2.249	−0.065	0.492	−1.541	−0.047	0.588
Household income	−2.790	−0.127	0.193	−2.846	−0.138	0.138
Number of pregnancies	−4.080	−0.131	0.295	−3.812	−0.128	0.288
Number of deliveries	−0.408	−0.108	0.836	−0.554	−0.019	0.875
Nutrient supplementation	0.735	0.024	0.848	0.534	0.025	0.766
Blood OCM indicators						
Serum folate	—	—	—	−0.072	−0.663	0.002*
RBC folate	−0.025	−0.330	0.006*	—	—	—
Serum choline	−0.259	−0.063	0.440	0.163	0.041	0.602
Serum betaine	0.024	0.191	0.837	−0.021	−0.090	0.837
Serum methionine	−0.013	−0.059	0.588	0.064	0.125	0.749
Serum vitamin B_2_	0.082	0.041	0.632	0.118	0.062	0.393
Serum vitamin B_6_	−0.056	−0.317	0.026*	−0.082	−0.476	0.039*
Serum vitamin B_12_	0.014	0.068	0.410	0.011	0.082	0.413
Plasma SAM	−0.050	−0.073	0.486	0.047	0.169	0.482
	Adjusted *R^2^* = 0.273, *p* < 0.05			Adjusted *R^2^* = 0.370, *p* < 0.05		

Variable values: Age (1: 18 ~ 29, 2: 30 ~ 34, 3: 35 ~ 46); Gestational weeks (1: 24 ~ 27, 2: 28 ~ 31); Educational level (1: ≤High school, 2: Junior college, 3: ≥Bachelor); Employment (1: employed, 2: unemployed); Household income (1: ≤6,000, 2: 6001 ~ 10,000, 3: ≥10,001); Number of pregnancies (1: 1, 2: ≥2); Number of delivery (1: 0, 2: ≥1); Nutrient supplementation (1: no supplement, 2: supplement with folic acid, 3: supplement without folic acid). *p < 0.05.

Additionally, multiple linear regression analysis of SAM (Table 7) indicated associations between blood betaine and vitamin B_6_ with plasma SAM. When RBC folate (model 1) was considered as the independent variable, plasma SAM exhibited positive associations with serum betaine (*β* = 0.610, *p* < 0.05), while displaying negative associations with serum vitamin B_6_ (*β* = −0.181, *p* < 0.05). These results suggested that higher betaine and lower vitamin B_6_ levels served as predictors of appropriate SAM levels.

**Table 7:** Multiple linear regression analysis of plasma SAM of pregnant women.

Independent variable	Model 1			Model 2		
	*B*	*β*	*p*	*B*	*β*	*p*
Age	41.264	−0.007	0.910	0.542	0.019	0.758
Gestational weeks	−0.221	0.035	0.570	1.252	0.023	0.700
Educational level	2.014	0.015	0.836	0.741	0.029	0.676
Employment	0.389	0.137	0.050*	6.375	0.134	0.042*
Household income	6.843	0.044	0.556	0.889	0.029	0.683
Number of pregnancies	1.393	−0.011	0.908	−0.747	−0.017	0.850
Number of deliveries	−0.482	0.048	0.608	1.819	0.042	0.649
Nutrient supplementation	2.168	−0.05	0.425	−1.550	−0.062	0.303
Blood OCM indicators						
Serum folate	—	—	—	−0.03	0.068	0.764
RBC folate	0.005	0.057	0.364	—	—	—
Serum choline	−0.41	−0.069	0.270	−0.455	−0.078	0.199
Serum betaine	0.960	0.610	<0.001*	0.907	0.638	<0.001*
Serum methionine	−0.367	−0.096	0.121	−0.395	−0.106	0.077
Serum vitamin B_2_	−0.248	−0.084	0.192	−0.300	−0.125	0.056
Serum vitamin B_6_	−0.451	−0.181	0.004*	−0.447	−0.202	0.001*
Serum vitamin B_12_	−0.019	−0.067	0.287	−0.015	−0.057	0.332
Serum tHcy	−0.067	−0.046	0.451	−0.066	−0.045	0.441
	Adjusted *R^2^* = 0.410, *p* < 0.001			Adjusted *R^2^* = 0.392, *P* < 0.001		

Variable values: Age (1: 18 ~ 29, 2: 30 ~ 34, 3: 35 ~ 46); Gestational weeks (1: 24 ~ 27, 2: 28 ~ 32); Educational level (1: ≤High school, 2: Junior college, 3: ≥Bachelor); Employment (1: employed, 2: unemployed); Household income (1: ≤6000, 2: 6001 ~ 10,000, 3: ≥10,001); Number of pregnancies (1: 1, 2: ≥2); Number of delivery (1: 0, 2: ≥1); Nutrient supplementation (1: no supplement, 2: supplement with folic acid, 3: supplement without folic acid). *p < 0.05.

## 4 Discussion

OCM plays a critical role in fetal growth and neurodevelopment by transferring methyl group to biomolecules. This process involves folate, methionine, choline and betaine acting as methyl donor nutrients, vitamin B_2_, B_6_, B_12_ acting as enzyme cofactors; and homocysteine and SAM, serving as functional intermediate metabolites. In the study, OCM nutrients and metabolites levels were measured in 397 Chinese women in mid-to-late pregnancy. The results revealed serum deficiencies of folate, vitamin B_6_, and B_12_ at 2.5, 1.3, and 8.3% respectively, with a concurrent incidence of 1.8% hyperhomocysteinemia. Within the folate cycle, RBC folate demonstrated a positive correlation with the cofactor vitamin B_12_. Regarding functional markers of OCM, the study identified serum and RBC folate and serum vitamin B_6_ as significant negative predictors of serum tHcy. Additionally, plasma SAM exhibited a positive association with serum betaine, while displaying a negative association with serum vitamin B_6_.Previous studies predominantly focused solely on folate status, particularly dietary folate intake and its relationship with health outcomes. Actually, dietary exposure could not reflect OCM metabolism precisely, in which the folate cycle interacts with the methionine cycle. Besides, OCM was related to not only the neural tube defects occurring in early pregnancy, but also the fetal physical and mental development, even childhood health. Therefore, it is imperative to explore the relationships of OCM during different trimesters with both maternal and offspring outcomes.

Since the 1990s, worldwide recommendations for folic acid supplementation during periconception have resulted in very high rates of periconceptional folic acid supplementation, with 71.0% of pregnant women in this study reported to have taken folic acid supplementation. Similarly, another Chinese rural cohort study showed a rate of 83.2% among pregnant women (21). However, folate deficiency remains prevalent. Studies showed 24% of North American women had folate deficiency in their first trimester (9), and 24% of Indian women had folate deficiency (24). In this study, there was 2.5% of serum folate deficiency during mid-to-late pregnancy. One plausible explanation for this finding is that folate metabolism is influenced not only by dietary folate intake (including folic acid supplementation) but also by metabolic cofactors such as vitamin B_2_, B_6_, and B_12_. In fact, this study reported a vitamin B_12_ deficiency rate of up to 8.3% and observed a positive correlation between RBC folate and vitamin B_12_ levels. A review indicated that vitamin B_12_ insufficiency affected 21, 19, and 29% of women in the first, second, and third trimesters, respectively, (29). Vitamin B_12_ deficiency disrupts the one-carbon cycle and impedes the conversion of 5-methyl tetrahydrofolate to tetrahydrofolate (30). Hence, it is imperative to improve not only folate but also metabolic cofactors (such as vitamin B_12_) throughout pregnancy.

Furthermore, aside from its involvement in neural tube differentiation in early pregnancy, OCM plays a role in mid-to-late fetal development and contributes to the occurrence of maternal anemia and hyperhomocysteinemia (HHcy). The high prevalence of OCM disturbance among mid-to-late pregnant women in this study underscores the importance of continuous folic acid supplementation throughout pregnancy, which has been recommended in Chinese Dietary Guidelines (2022) (31). Moreover, the study highlighted a significant correlation between the sociodemographic characteristics of pregnant women and their OCM levels. Specifically, educational attainment and household income exhibited a positive association with the concentrations of maternal RBC folate, serum choline and vitamin B_2_. This aligns with prevailing research indicating that women with higher levels of education and socioeconomic status (SES) tend to adhere more closely to dietary guidelines, thereby meeting essential nutrient requirements (32, 33). Conversely, lower educational attainment and SES may predispose individuals to unhealthy lifestyles characterized by dietary imbalances, obesity, and smoking (32, 33). Additionally, women belonging to lower educational level and SES often have infrequent antenatal visits, which diminishes their opportunities for dietary assessments, necessary adjustments, and adequate supplementation (32, 33). While some studies suggest that older women are more likely to adhere to healthier diets (32), animal research indicated that folate-modulated DNA methylation decreased with age (34, 35). This could explain the notably lower serum methionine levels observed in pregnant women aged 35 and above in this study. The negative correlation between multiparity and OCM levels might also be attributed to advanced age and postpartum recovery (32, 33). However, it is noteworthy that the number of pregnancies/deliveries did not exhibit a significant relationship with tHcy and SAM in multiple regression analysis. This observation highlights the need for increased attention to this demographic group.

Homocysteine serves as the principal functional biomarker of OCM, and undergoes catabolism via remethylation and transsulfuration pathways, where folate acts as a methyl donor and vitamins B_6_ and B_12_ function as enzyme cofactors. Maternal Hcy concentration decreased from early to mid-gestation and increased from mid-to-late gestation in a normal pregnancy (36). Clinical studies have highlighted the potential impact of elevated maternal homocysteine during pregnancy on fetal cognitive decline (20) and Down’s syndrome (22). Insights from the Ottawa and Kingston birth cohort suggest an independent effect of early to mid-pregnancy elevated maternal Hcy on placenta-mediated pregnancy complications, even on children’s cardiovascular-metabolic diseases after delivery (13, 36, 37). In this study, 1.8% of mid-to-late pregnant women exhibited HHcy, with adequate blood folate and vitamin B_6_ identified as protective factors against HHcy. Other studies have similarly indicated that sufficient dietary intake and serum concentrations of folate and other B-vitamins are crucial to maintaining normal Hcy levels (38, 39). Moreover, genetic variations in metabolic enzyme-encoding genes could contribute to elevated homocysteine levels (38, 40, 41), though this aspect was not examined in the study. Nevertheless, to mitigate the risk of HHcy and promote maternal-offspring health, attention to folate and other B-vitamin status remains critical.

SAM provides methyl to DNA, proteins and lipids directly within OCM, as the single direct factor associated with most biomethylation reactions (42), while folate and choline are recognized as primary methyl donors. A decrease in SAM levels strongly correlates with reduced OCM function. The level of SAM is noteworthy, but studies focused on SAM are relatively scarce, and definitive cutoff levels are yet to be established. In this study, the median SAM of pregnant women was 52.50 (*P*_25_ ~ *P*_75_: 43.50 ~ 65.60) nmol/L, and a positive correlation between SAM and betaine levels was found. A case–control study in China showed that SAM concentration was 49.9(±3.0) nmol/L in women with neural tube defects-affected children, and 52.8(±3.2) nmol/L in women with healthy children (43). Interestingly, the study showed plasma SAM was negatively correlated with serum vitamin B_6_, which is not directly involved in SAM or choline-betaine metabolism but rather in homocysteine catabolism. One possible explanation is that vitamin B_6_ deficiency can lead to the accumulation of Hcy, resulting in upstream S-adenosylhomocysteine (SAH) elevation and subsequent SAM elevation through feedback regulation. An animal study showed that with age, folate deficiency alters the methylation index reflected by the SAM/SAH ratio, leading to a decrease in overall methylation (44). In this study, SAM concentration was measured while SAH was ignored. Accidental errors could be present in the results, and SAH would be explored in subsequent studies. Additionally, the cutoff level of SAM has not been harmonized and more attention should be paid to defining the cutoff levels of SAM to strengthen the study’s findings.

Furthermore, attention should be directed toward choline-betaine metabolism. This study noted a positive correlation between serum folate levels and serum choline and betaine, indicating a potential interaction between these two methyl donor pathways.

This study comprehensively illustrates the intricate interplay between folate and other nutrients in OCM, emphasizing that folate status alone is insufficient. It underscores the necessity of evaluating additional OCM markers, including vitamins B_2_, B_6_, B_12_, as well as Hcy, SAM, and choline, during routine prenatal screenings. This is particularly crucial for women with lower educational backgrounds. When warranted, these B-vitamin levels can be optimized through a balanced diet and targeted supplementation. The alarming prevalence of OCM disturbances among women in the mid-to-late stages of pregnancy underscore the critical importance of maintaining consistent B-vitamin supplementation throughout the entire gestational period.

## 5 Conclusion

The study revealed an imbalance in blood OCM during mid-to-late pregnancy, characterized by lower folate, vitamin B_6_, and B_12_ levels alongside elevated tHcy levels. Adequate folate and vitamin B_6_ emerged as significant predictors of lower tHcy levels, and a positive association was noted between serum betaine and plasma SAM.

### 5.1 Limitations

OCM encompasses not only a range of methyl donor nutrients but also metabolic enzymes, including methyl tetrahydrofolate reductase (MTHFR) and cystathionine-β-synthase (CBS), whose activities are intricately linked by genetic variations and expression levels. Nevertheless, the current study did not delve into the genetic factors at play. Furthermore, while most OCM nutrients and metabolites were examined, certain indicators like S-adenosyl homocysteine and dimethylglycine were overlooked. Except for the folic acid supplement, information about other B-vitamin supplementation (B_2_, B_6_, B_12_) was not included. Furthermore, it is well established that OCM status undergoes significant variations across different trimesters of pregnancy. However, this study did not explore the nuances of early pregnancy. It is imperative that future research addresses these noted limitations to gain a more profound understanding of the profound implications of maternal OCM on both offspring development and maternal health outcomes.

## Data availability statement

The original contributions presented in the study are included in the article/supplementary materials, further inquiries can be directed to the corresponding author.

## Ethics statement

The studies involving humans were approved by the Peking University Institutional Review Board (Beijing, China, approval number IRB00001052-19145), and conducted according to the Declaration of Helsinki. The studies were conducted in accordance with the local legislation and institutional requirements. Written informed consent for participation in this study was provided by the participants’ legal guardians/next of kin.

## Author contributions

RZ: Data curation, Formal analysis, Investigation, Writing – original draft, Writing – review & editing. XW: Investigation, Writing – review & editing. LL: Investigation, Resources, Writing – review & editing. RH: Investigation, Resources, Writing – review & editing. YT: Investigation, Resources, Writing – review & editing. LP: Investigation, Resources, Supervision, Writing – review & editing. XZ: Investigation, Resources, Supervision, Writing – review & editing. WJ: Investigation, Resources, Writing – review & editing. WL: Investigation, Resources, Writing – review & editing. LD: Investigation, Resources, Writing – review & editing. WZ: Conceptualization, Investigation, Methodology, Project administration, Resources, Writing – review & editing.
